# Activation of Wnt/β-Catenin Pathway in Monocytes Derived from Chronic Kidney Disease Patients

**DOI:** 10.1371/journal.pone.0068937

**Published:** 2013-07-23

**Authors:** Heevy Abdulkareem Musa Al-Chaqmaqchi, Ali Moshfegh, Elham Dadfar, Josefin Paulsson, Moustapha Hassan, Stefan H. Jacobson, Joachim Lundahl

**Affiliations:** 1 Department of Laboratory Medicine, Division of Experimental Cancer Medicine-Clinical Research Center, Karolinska Institutet, Stockholm, Sweden; 2 Department of Oncology and Pathology, Cancer Center Karolinska (CCK), Karolinska Hospital and Karolinska Institutet, Stockholm, Sweden; 3 Department of Clinical Immunology and Transfusion medicine, Karolinska University Hospital, Karolinska Institutet, Stockholm, Sweden; 4 Clinical Research Centre (KFC, Novum), Karolinska University Hospital-Huddinge, Stockholm, Sweden; 5 Division of Nephrology, Department of Clinical Sciences, Karolinska Institutet, Danderyd University Hospital, Stockholm, Sweden; Institut national de la santé et de la recherche médicale (INSERM), France

## Abstract

Patients with chronic kidney disease (CKD) have significantly increased morbidity and mortality resulting from infections and cardiovascular diseases. Since monocytes play an essential role in host immunity, this study was directed to explore the gene expression profile in order to identify differences in activated pathways in monocytes relevant to the pathophysiology of atherosclerosis and increased susceptibility to infections. Monocytes from CKD patients (stages 4 and 5, estimated GFR <20 ml/min/1.73 m^2^) and healthy donors were collected from peripheral blood. Microarray gene expression profile was performed and data were interpreted by GeneSpring software and by PANTHER tool. Western blot was done to validate the pathway members. The results demonstrated that 600 and 272 genes were differentially up- and down regulated respectively in the patient group. Pathways involved in the inflammatory response were highly expressed and the Wnt/β-catenin signaling pathway was the most significant pathway expressed in the patient group. Since this pathway has been attributed to a variety of inflammatory manifestations, the current findings may contribute to dysfunctional monocytes in CKD patients. Strategies to interfere with this pathway may improve host immunity and prevent cardiovascular complications in CKD patients.

## Introduction

Patients with CKD are characterized by dysfunction in both innate and adaptive immunity. Therefore, they are more susceptible to infections and have a low response to vaccination.[1]–[4] Another feature of CKD is the presence of low grade systemic inflammation, which predisposes the patient for disease progression and manifestation of cardiovascular disease [5], [6].

Monocytes play a pivotal role in both innate and adaptive immunity and constitute 5–10% of peripheral blood leukocytes. [7] They originate in the bone marrow and are released into the peripheral circulation, circulating for several days before entering tissues where they finally differentiate into macrophages or dendritic cells. [8] This process involves several adherence-detachment events mediated by selectins and integrins on monocytes and endothelial cells. [9] Monocytes are provided with a large number of scavenger receptors that recognize various microorganisms, and activated monocytes can produce large amounts of cytokines such as TNF-α, IL-6, IL-10, CXCL8, vascular endothelial growth factor (VEGF) and proteolytic enzymes [8].

Several reports have demonstrated alterations in adhesion and migratory capabilities in monocytes from CKD patients, [10], [11] and several studies have shown that the adhesion competence of monocytes to vascular endothelium is increased in CKD patients. [12] These characteristics may predispose this patient group to increased frequency of atherosclerotic complications [13].

Apart from the abovementioned changes in monocyte features, little is known about the genetic changes that occur in patients with advanced CKD. [14] Global gene expression profiles provide wide-ranging information about alterations that occur in gene expression in relation to different diseases. We did microarray gene expression profiles on monocytes collected from the peripheral blood in CKD patients and healthy individuals. Based on the clinical manifestations of the disease, we hypothesized that pathways relevant for infection and inflammation were up regulated in monocytes from CKD patients. We focused on the Wnt/β-catenin signaling pathway since abnormal Wnt signaling has been associated with many human diseases ranging from cancer and inflammation to degenerative diseases [15], [16].

## Materials and Methods

### Study Population

The study population consisted of fourteen patients with a median age of 59 years (50–72) suffering from CKD stage 4–5 (estimated GFR<20 ml/min/1.73 m^2^ according to Cockroft and Gault equation) and ten healthy subjects. The healthy subjects were age- and sex matched with the patients. The renal diagnoses were the following: nephrosclerosis, autosomal dominant polycystic kidney disease and renal failure of unknown origin. Cells from three patients and three healthy subjects were used for Affymetrix analysis. Western blot analysis was run for all samples. None of the patients or healthy subjects had diabetes mellitus or any clinical signs of active inflammatory disease. They were not receiving antibiotics, corticosteroids or non-steroidal anti-inflammatory agents. Written informed consent was obtained from all participants. The study was approved by the ethics committee of the Karolinska University Hospital, Stockholm, Sweden.

### Collection and Purification of Monocytes from the Peripheral Circulation

50–60 ml of peripheral blood was collected in 9 ml tubes containing 135USP U sodium heparin (Venosafe: Terumo Europe, Leuven, Belgium). Monocytes were isolated from the peripheral blood by density centrifugation. Briefly, blood was diluted 1∶1 with RPMI 1640. The diluted blood was then layered on 25 ml Ficoll Paque (Pharmacia Biotech, Uppsala, Sweden) and centrifuged at 400×g in room temperature for 30 minutes. A monocyte rich white color band was collected. A positive selection of monocytes was performed by incubating the monocytes with anti-CD14 coupled to MACS beads (Miltenyi Biotec, Auburn, California, USA). The cells were then loaded onto a MidiMACS column and the CD14 positive monocytes were collected.

### Purity of Monocytes

The purity of the monocyte fraction was analyzed by flow cytometry (forward and side scatter properties), staining with CD14–ECD (Beckman Coulter, Marseille, France) and using isotype mouse IgG2 (Beckman Coulter, Marseille, France). Only fractions above 95% purity were used for further experiments. Purified monocytes were either cryopreserved in Qiagen RLT-buffer at −70°C for subsequent RNA extraction or lysed in Western blot lysing buffer and subsequently used for protein preparation.

### RNA Extraction

Total RNA from purified monocytes was extracted with the Qiagen RNeasy kit (WVR, Stockholm, Sweden) according to the manufacturer’s instructions. The integrity of the extracted RNA was confirmed by nano-drop ND-1000 UV–Vis Spectrophotometer (NanoDrop Technologies,Wilmington, DE) with extracts exhibiting absorbance in a ratio of 1.99–2.0 at 260/280 nm regarded as being of acceptable purity, and agarose gel was run to confirm purity of RNA.

### Sample Preparation for Affymetrix Microarray Gene Expression Analysis

Double-stranded cDNA was synthesized with 50 ng of total RNA using the SuperScript Choice system (Invitrogen Inc). T7-(dT24) oligomer was used for priming the first-strand cDNA synthesis. The resultant cDNA was purified using the Sample clean up kit (Affymetrix Inc). The cDNA pellet was collected and dissolved in an appropriate volume. Using cDNA as the template, cRNA was synthesized using an In-vitro transcription (IVT) kit (Affymetrix Inc). IVT reactions were carried out at 37°C for 16 hours and the labeled cRNA was purified using the Sample clean up kit (Affymetrix Inc). The cRNA was fragmented in a fragmentation buffer (40 mmol/l Tris-acetate, pH 8.1, 100 mmol/l KOAc, 30 mmol/l MgOAc) for 35 min at 94°C. Fragmented cRNA (15 µg/probe array) was hybridized with the human U133A GeneChip arrays, containing 22 283 sets of probes for approximately 17 000 genes, at 45°C for 18 hours in a hybridization oven with constant rotation (60 rpm). The chips were washed and stained using the Affymetrix fluidics stations. Staining was performed using Streptavidin phycoerythrin conjugate (SAPE; Molecular Probes, Eugene, OR, USA), followed by the addition of a biotinylated anti-streptavidin antibody (Vector Laboratories, Burlingame, CA, USA), and finally with Streptavidin phycoerythrin conjugate. Probe arrays were scanned using fluorometric scanners (Affymetrix Scanner). The scanned images were inspected and analyzed using established quality control criteria. The arrays were first analyzed using GeneChip operating software (GCOS 1.4 Affymetrix). A quantitative signal and a qualitative detection call were generated for each sample and transcript. Data file were subsequently analyzed utilizing the GeneSpring GX11.5 (Silicon Genetics, CA, USA).

### Western Blot

Whole cell lysate was prepared using modified radioimmunoprecipitation assay (modified RIPA) buffer. Briefly, isolated purified monocytes were washed with cold PBS and lysed in modified RIPA containing 150 mM NaCL, 50 mM Tris-HCL Ph 7.5, 1 mM EDTA, 0.1% Triton X-100, 1% CHAPS with proteinase inhibitors (Complete, Boehringer Mannheim, Germany), and phosphatase inhibitor (Phos stop, Roche). Two cycles of freezing and thawing on dry ice with ethanol were performed, and the sample was then left in ice for 30 min. Debris were removed by centrifugation at 10000 rpm for 10 min at 4°C and the supernatants were collected. After adjustment of protein concentration by Pierce BCA Protein Assay Kit (Thermo Scientific, Rockford, IL, USA) with a spectrometer at 540 nm, the sample was stored in 4 x sample buffer in a concentration of 2.5 ug/ml. Sample buffer containing 1 M Tris Ph 6.8, β mercaptoethanol, glycerol, bromophenol blue, (sodium dodecyl sulfate) SDS. The lysates in sample buffer were boiled in 95°C for 5 min and separated by 10% Bis-Tris Gel (Nu PAGE ®, Novex, Life technologies, USA). Gels were blotted on a polyvinylidene fluoride membrane (Immobilon P, Millipore, Bedford, MA). The membranes were blocked in 5% milk followed by incubation with antibodies against β-catenin, GSKβ, PGSKβ, DVL1, CK1ε and Tubulin. After washing, the membranes were incubated with secondary antibody conjugated horseradish peroxidase followed by enhanced chemiluminescence (ECL) detection (ECL Plus, Amersham Pharmacia, Uppsala, Sweden).

### Quantification of Western Blot Result

For quantification of Western blot results, Image J software was used. Tubulin was used as house-keeping protein to normalize the results. For statistical analysis of western blot unpaired t-test was used.

### Microarray Gene Expression Data Analysis

The expression analysis file created from each sample (chip) scanning was imported to GeneSpring™ software version GX11.5 (Silicon Genetics, CA, USA) for further data characterization. Gene expression data were RMA (Robust multi-array average) normalized. Unpaired T-test with Benjamin-Hochberg discovery rate was used as statics analysis according to Genspring software protocol. [17] The data sets were then assigned to two groups for monocyte experiments in healthy subjects and in patients. List were acquired of genes that were either induced or suppressed >1.5 fold between patients with CKD stage 4–5 and healthy subjects. For pathway analysis, the PANTHER classification system was used. Microarray data were submitted to the Gene Expression Omnibus (http://www.ncbi.nlm.nih.gov/geo/) with the accession number GSE43484.

## Results

### Quality of Cell and RNA Preparation

The preparations used for gene profiling were of >95% purity as determined phenotypically. The microarray analysis showed no detectable levels of lineage specific transcripts for non-monocyte/macrophage cell lineages (Table 1). The monocyte/macrophage lineage specific transcript (M-CSFR) was expressed while no expression of markers for T cells (TCR), endothelial cells (VEGFR & VECAM1), polymorphonuclear cells (G-CSFR) or eosinophils/basophils (IL-5RA) were detected from purified monocytes in peripheral circulation. These findings reduce the probability that one or more of the identified genes have arisen from a trace population of contaminating cells.

**Table 1:** Detection of lineage specific genes in monocyte purifications.

Gene Name	Cell-Type Expression Lineage Specific genes	Detectable transcripts from purified monocyte preparations
G-CSFR	PMN	No
IL-5RA	Eosinophil/basophil	No
M-CSFR	Monocyte/macrophage	yes
TCR	T cell	No
VEGFR	Endothelial cells	No
VECAM1	Endothelial cells	No

### Global Comparison of Genes from CKD Derived Monocytes and Healthy Donor Monocytes

**Figure 1:**
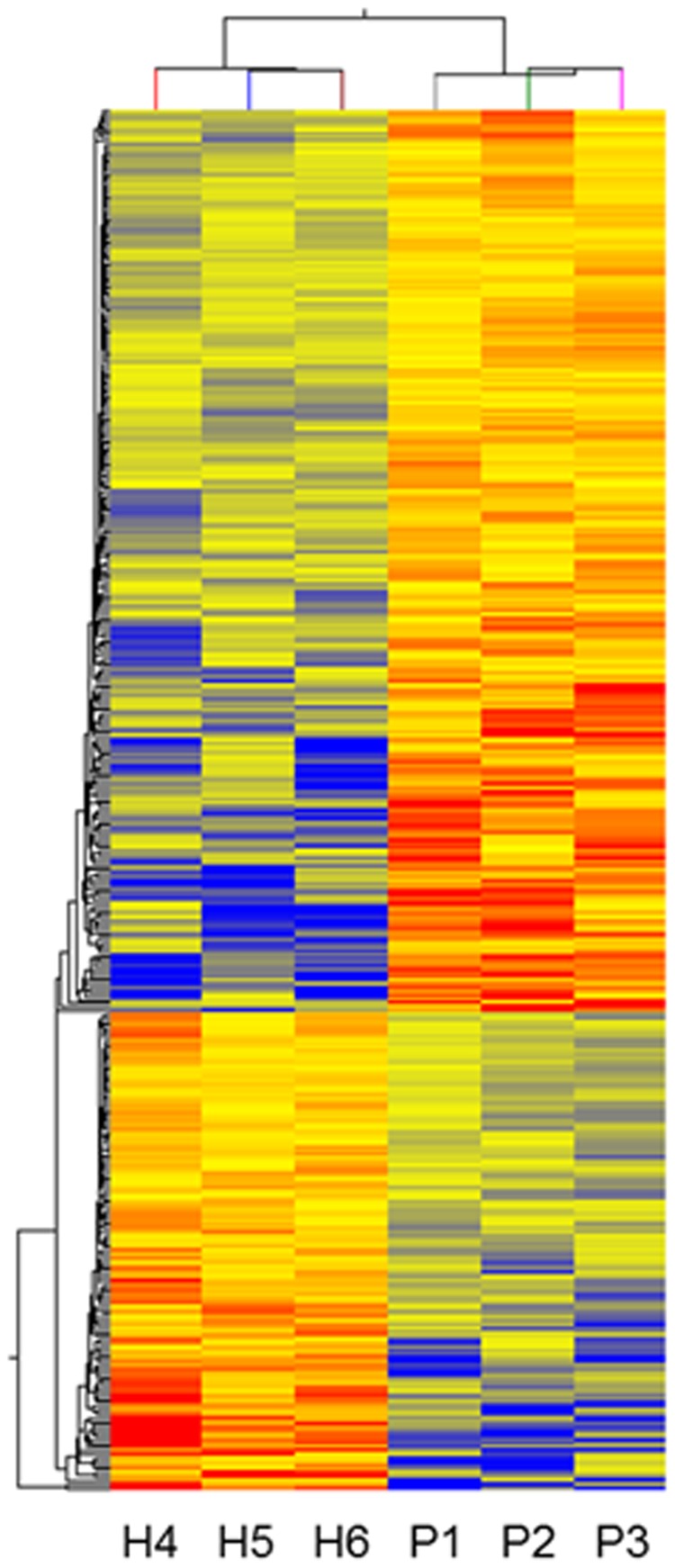
Gene expression pattern in peripheral monocytes from three healthy subjects (H4, H5, H6) and three patients with CKD stages 4 and 5 (eGFR <20 ml/min/1.73 m^2^) (P1, P2, P3). The gene expression intensity is presented in red (high expression intensity) and blue (low expression intensity).

In this study approximately 17 000 genes were compared for differential expression in monocytes. In order to find those probes with the highest difference between the two groups as well as with statistical significance, we employed a t-test (set to *P*<0.05 and a fold change of 1.5). A 2-way unsupervised hierarchical clustering with an average linkage rule and a Euclidean centered correlation is shown in Figure 1. The unsupervised analysis clearly separates the two groups of healthy individuals and patients, demonstrating their overall dissimilarity. As illustrated, 600 genes were up regulated and 272 down regulated in CKD patients. Full lists of up and down regulated genes are provided as supplementary information to this paper as Table S1 and Table S2.

### Pathway Classification of up Regulated Genes in CKD Patients

Pathway analysis by PANTHER tool showed that the Wnt signaling pathway contains the largest number of up regulated genes, which are 15. Other pathways were inflammation mediated by chemokine and cytokine signaling, TGF-β pathway signaling, integrin pathway signaling and interleukin pathway signaling. Table 2 shows the pathways with the largest number of up regulated genes in the CKD group. Table 3, Table 4, Table 5, Table 6 and Table 7 shows the genes involved in each of these pathways.

**Table 2:** Pathways up regulated in monocytes collected from patients with CKD stages 4 and 5.

Name of pathway	No. of genes
Wnt signaling pathway	15
Inflammation mediated by chemokine and cytokine signaling pathway	12
TGF β signaling pathway	7
Integrin signaling pathway	6
interleukin signaling pathway	5

**Table 3:** Wnt signaling pathway.

Mapped IDs	Fold change	Gene name
ANKRD6	1.6	Ankyrin repeat domain-containing protein 6
CDH15	1.7	Cadherin-15
CDH19	4.5	Cadherin-19
CDH8	3.2	Cadherin-8
DKK2	5	Dickkopf-related protein 2
EDN1	2.1	Big endothelin-1
FZD4	1.8	Frizzled-4
MYH8	1.9	Myosin-8
NFATC4	1.9	Nuclear factor of activated T-cells, cytoplasmic 4
PCDHA5	5.9	Protocadherin alpha-5
PCDHA9	2	Protocadherin alpha-9
PPARD	3.8	Peroxisome proliferator-activated receptor delta
PPP2R5C	1.9	Serine/threonine-protein phosphatase 2 regulatory subunit gamma isoform
TCF3	2.2	Transcription factor 7-like 1
WNT5A	5.6	Protein Wnt-5a

**Table 4:** Inflammation mediated by chemokine and cytokine signaling pathway.

Mapped IDs	Fold changes	Gene name
C3AR1	1.8	C3a anaphylatoxin chemotactic receptor
CCL21	1.5	C-C motif chemokine 21
CCR3	3.3	C-C chemokine receptor type 3
CISH	1.7	Cytokine-inducible SH2-containing protein
CXCR5	1.8	C-X-C chemokine receptor type 5
CXCR6	1.8	C-X-C chemokine receptor type 6
EVI1	2.7	Ecotropic virus integration site 1 protein homolog
IL6	1.5	Interleukin-6
MYH11	2.2	Myosin-11
MYH8	1.9	Myosin-8
NFATC4	1.9	Nuclear factor of activated T-cells, cytoplasmic 4
PAK7	3.4	Serine/threonine-protein kinase PAK 7

**Table 5:** TGFβ signaling pathway.

Mapped IDs	Fold changes	Gene name
BMP1	1.8	Bone morphogenetic protein 1
BMP6	3.0	Bone morphogenetic protein 6
FKBP1B	1.5	Peptidyl-prolyl cis-trans isomerase
FOXN3	1.6	Forkhead box protein N3
LEFTY1	1.5	Left-right determination factor 1
SMURF1	1.6	E3 ubiquitin-protein ligase
TLL2	1.8	Tolloid-like protein 2

**Table 6:** Integrin signaling pathway.

Mapped IDs	Fold changes	Gene name
ACTN3	2.3	Alpha-actinin-3
ARHGAP10	1.8	Rho GTPase-activating protein 10
COL4A3	2.2	Tumstatin
ELMO2	1.9	Engulfment and cell motilityprotein 2
ITGA6	4.3	Integrin alpha-6 light chain
LAMA2	7.7	Laminin subunit alpha-2

**Table 7:** Interleukin signaling pathway.

Mappeed IDs	Fold changes	Gene name
BAD	1.8	Bcl2 antagonist of cell death
FOXN3	1.6	Forkhead box protein N3
IL11	1.6	Interleukin-11
IL6	1.5	Interleukin-6
IRS2	2.0	Insulin receptor substrate 2

### Wnt/β-catenin Pathway is Activated in CKD Monocytes

Different genes involved in the Wnt signaling pathway were up regulated in microarray gene expression results (Table 3). Follow-up experiments by Western blot to confirm the presence of proteins involved in this pathway were performed.

**Figure 2:**
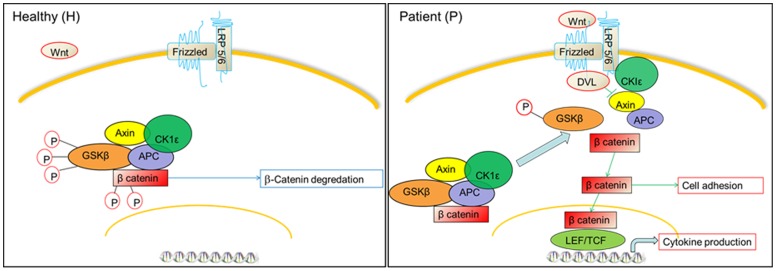
Schematic view for Wnt/β-catenin signaling shows the expression of genes and proteins involved in Wnt/β-catenin signaling in both CKD patients (P) and healthy subjects (H).

Figure 2 shows the Wnt/β-catenin pathway (canonical pathway) and all known proteins involved. In its inactive state, i.e. in healthy control (H), β-catenin exists within a protein complex consisting of Axin, APC (adenomatous polyposis coli), GSKβ (Glycogen synthase kinase) and CK1ε (Casein Kinase). When there is absence of signal through the receptor frizzled, this leads to phosphorylation and destruction of β-catenin, and prevents its accumulation and passing to the nucleus. [18] In the patient group (P), activation of the canonical pathway ensues when Wnt proteins interact with specific cell surface receptor complexes that consist of members of the Frizzled family and low-density lipoprotein receptor-related protein 5 or 6 (LRP5 or LRP6). This triggers phosphorylation of dishevelled proteins (DVL) and promotes their interaction with the Frizzled proteins. [19], [20] The DVL/receptor complexes facilitate phosphorylation of the LRP6 intracellular tails by CK1ε. As a consequence, Axin is recruited to this receptor complex and the degradation of β-catenin by proteasome is blocked. This allows β-catenin to accumulate and enter the nucleus, where it interacts with members of the Tcf/Lef family and converts them into potent transcriptional activators [15], [21]–[23].

**Figure 3:**
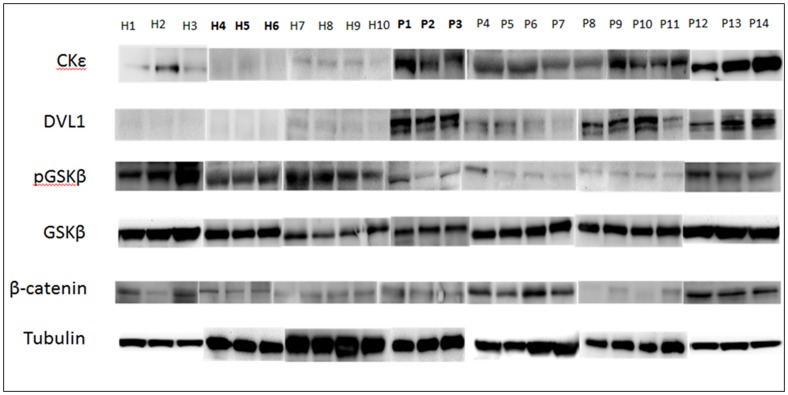
Western blot of CK1ε, DVL1, PGSKβ, GSKβ, β-catenin, and Tubulin from ten healthy subjects (H) and fourteen CKD patients (P). The sample name which is in bold corresponds to the sample used in microarray.

**Figure 4:**
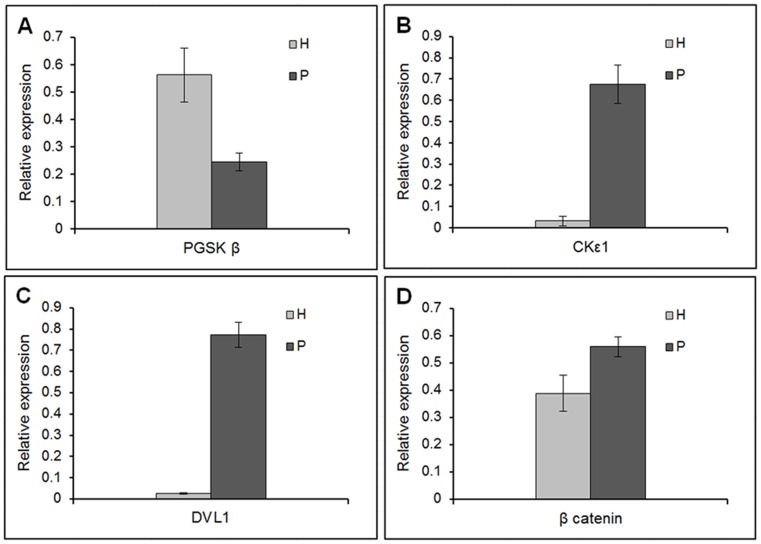
Densitometry of Western blot from fourteen patients and ten healthy Tubulin was used to normalize the results of β-catenin, DVL1 and CK1ε while GSKβ were used to normalize PGSKβ A) PGSK (*P<0.01*), B) CK1ε (*P<0.0001*), C) DVL1 (*P<0.00001*) and D) β-catenin (*P<0.05*d).

Figures 3 show the Western blot for all the samples. P1, P2, P3, H4, H5, and H6 in the western blot correspond to the same patient and healthy samples used in the microarray gene expression analysis. Figure 4 show the densitometry of Western blot for quantification of β-catenin, GSKβ, PGSKβ, DVL1 and CK1ε. It is evident that the protein concentration is different between the CKD patients and the healthy subjects. β -catenin, the main protein in this pathway, was significantly more expressed in the patient group (*P<*0.05). There were no differences in total GSKβ between the healthy subjects and the patient group, but the phosphorylated GSKβ (P GSKβ) was significantly more expressed in healthy subjects than in patients (*P<*0.01). DVL1 and CK1ε were significantly more expressed in patients than in healthy subjects (*P<*0.00001 and *P<*0.0001, respectively). Table 8 shows the fold changes of the proteins in the patient relative to healthy. β-catenin, DVL1 and CK1ε were increased by 1.4, 28.6 and 21.3 fold changes in the patient relative to healthy. While PGSKβ were down regulated in the patient by 2.3 fold changes relative to healthy.

**Table 8:** Fold changes of the proteins expressed by Western blots in patients compared to healthy controls.

Protein name	Protein symbol	Fold changes	P-value
**Beta catenin**	β catenin	1.4	<0.05
**Phosphorylated Glycogen synthetase kinase beta**	PGSK β	−2.3	<0.01
**Dishevelled 1**	DVL1	28.6	<0.00001
**Casein kinase epsilon1**	CKε1	21.3	<0.0001

## Discussion

The present study presents data on the gene expression pattern of peripheral monocytes from patients with CKD stage 4–5. Genes involved in the Wnt signaling pathway showed significantly higher expression in CKD patients with a parallel up regulation of pathways related to inflammation.

Monocytes play an essential role in the human innate and adaptive immune response to infection, and CKD patients have increased susceptibility to infections. One important contributing factor is dysfunctional monocytes and antigen presenting cells. Many pathways related to the inflammatory response and cell adhesion were up regulated in patients with advanced CKD. This finding, together with activated chemokine and cytokine signaling, supports the view that these patients are in a state of chronic systemic inflammation, [6], [24], [25] involving dysregulated monocytes.[26]–[28] Characteristically, these patients have increased serum cytokine levels of IL-1, IL-6 and TNFα, which are associated with increased risk of mortality [25].

Our main observation was that key genes involved in the Wnt signaling pathway were significantly up regulated. Wnts constitute a large group of 19 secreted lipid modified glycoproteins and 12 frizzled receptors in vertebrates. Both receptor and ligand are highly conserved during evolution, and cross reactivity between them is very common [15], [16], [29]. This pathway plays an essential role in embryonic development, and produces important signaling molecules involved in adhesion processes including cell proliferation, differentiation, polarity, migration and invasion. [30] It has been linked to different inflammatory and degenerative diseases [31].

Our gene expression data demonstrated an up regulation of FZD4 and Wnt5a, which are important proteins in the Wnt signaling pathway, in CKD patients. Based on the observation by Pereira and colleagues, Wnt5a expression is increased in the sera of patients suffering from severe sepsis. [32] Wnt signaling has also been implicated in a number of chronic inflammatory diseases such as rheumatoid arthritis and atherosclerosis. [31], [33], [34] However; the causal link between Wnt function and etiology of the inflammatory disease has not been fully established.

The canonical Wnt pathway strictly controls the levels of a cytoplasmic protein known as β–catenin, which plays a crucial role in both cell adhesion and activation of Wnt target genes in the nucleus. In the absence of a Wnt signal, β-catenin is efficiently captured by a scaffold protein termed Axin, which is present within a protein complex (referred to as the destruction complex) that also connects adenomatous polyposis coli (APC) to glycogen synthase kinase (GSK)-3. Rapid activation of the canonical pathway occurs when Wnt proteins interact with specific cell surface receptor complexes comprising members of the Frizzled family. This triggers the phosphorylation of DVL proteins and promotes their interaction with the Frizzled proteins. The resulting DVL/receptor complexes are thought to stimulate the formation of LRP6 aggregates at the membrane, which facilitates the phosphorylation of the LRP6 intracellular tails by the CK1ε. As a consequence, Axin is recruited to this receptor complex and the proteasomal degradation of β-catenin is blocked.

Our subsequent experiments demonstrated increased protein levels of β–catenin, DVL1 and CK1ε, and decreased levels of phosphorylated GSKβ. This further confirmed activation of the canonical β-catenin pathway in CKD patients. We interpret these data to mean that β-catenin destruction is prevented. It accumulates in the cytoplasm and passes to the nucleus, where the protein exerts crucial effects on Wnt target genes involved in cell adhesion and activation [35].

CKD patients are in a chronic inflammatory state which is reflected by increased serum level IL-6 production. This is of specific interest since high IL-6 levels have been linked to increased production of Wnt signaling proteins. [36], [37] Moreover; Wnt5a in turn up regulate expression of proinflammatory gene IL-6. [38] Wnt5a is expressed during monocyte differentiation to dendritic cells, [39], [40] and its expression is known to result in more tolerant dendritic cells less responsive to infections. [41] Moreover, the Wnt/β-catenin pathway leads to increased monocyte adhesion to the endothelium and decreased migration through the endothelium. [42], [43] Wnt5a induces endothelial inflammation, [44] and is present in atherosclerotic lesions in humans. [45] Collectively, these data indicate that an up regulated Wnt pathway may impact the immunological status of CKD patients.

To summarize, our results demonstrate a different monocyte gene profile with a significant activation of the Wnt/β-catenin pathway in CKD stage 4–5 patients. Since this pathway is linked to dysregulation of monocyte adhesion, migration and inflammatory status, members of this pathway can be promising targets in improving immune status and preventing cardiovascular complications in patients with advanced CKD.

## Supporting Information

Table S1 **Down-regulated genes in patients (P) versus healthy (H) 1.5 fold changes.**
(XLSX)

Table S2 **Up-regulated genes in patients (P) versus healthy (H) 1.5 fold changes.**
(XLSX)

## References

[1] Cohen G, Haag-Weber M, Horl WH (1997) Immune dysfunction in uremia. Kidney Int Suppl 62: S79–82. 9350688

[2] Hauser AB, Stinghen AE, Kato S, Bucharles S, Aita C, et al (2008) Characteristics and causes of immune dysfunction related to uremia and dialysis. Perit Dial Int 28 Suppl 3S183–187. 18552253

[3] Haag-Weber M, Horl WH (1996) Dysfunction of polymorphonuclear leukocytes in uremia. Semin Nephrol 16: 192–201. 8734462

[4] Kato S, Chmielewski M, Honda H, Pecoits-Filho R, Matsuo S, et al (2008) Aspects of immune dysfunction in end-stage renal disease. Clin J Am Soc Nephrol 3: 1526–1533. 18701615 10.2215/CJN.00950208 PMC4571158

[5] Amann K, Tyralla K, Gross ML, Eifert T, Adamczak M, et al (2003) Special characteristics of atherosclerosis in chronic renal failure. Clin Nephrol 60 Suppl 1S13–21. 12940530

[6] Yilmaz MI, Carrero JJ, Axelsson J, Lindholm B, Stenvinkel P (2007) Low-grade inflammation in chronic kidney disease patients before the start of renal replacement therapy: sources and consequences. Clin Nephrol 68: 1–9. 17703829 10.5414/cnp68001

[7] Leon B, Lopez-Bravo M, Ardavin C (2005) Monocyte-derived dendritic cells. Semin Immunol 17: 313–318. 15955712 10.1016/j.smim.2005.05.013

[8] Rees AJ (2010) Monocyte and macrophage biology: an overview. Semin Nephrol 30: 216–233. 20620668 10.1016/j.semnephrol.2010.03.002

[9] Worthylake RA, Burridge K (2001) Leukocyte transendothelial migration: orchestrating the underlying molecular machinery. Curr Opin Cell Biol 13: 569–577. 11544025 10.1016/s0955-0674(00)00253-2

[10] Wedepohl S, Beceren-Braun F, Riese S, Buscher K, Enders S, et al (2012) L-Selectin - a dynamic regulator of leukocyte migration. Eur J Cell Biol 91: 257–264. 21546114 10.1016/j.ejcb.2011.02.007

[11] Faure V, Cerini C, Paul P, Berland Y, Dignat-George F, et al (2006) The uremic solute p-cresol decreases leukocyte transendothelial migration in vitro. Int Immunol 18: 1453–1459. 16954166 10.1093/intimm/dxl077

[12] Ramirez R, Carracedo J, Merino A, Soriano S, Ojeda R, et al (2011) CD14+CD16+ monocytes from chronic kidney disease patients exhibit increased adhesion ability to endothelial cells. Contrib Nephrol 171: 57–61. 21625090 10.1159/000327134

[13] Osterud B, Bjorklid E (2003) Role of monocytes in atherogenesis. Physiol Rev 83: 1069–1112. 14506301 10.1152/physrev.00005.2003

[14] Alcorta D, Preston G, Munger W, Sullivan P, Yang JJ, et al (2002) Microarray studies of gene expression in circulating leukocytes in kidney diseases. Exp Nephrol 10: 139–149. 11937761 10.1159/000049909

[15] Logan CY, Nusse R (2004) The Wnt signaling pathway in development and disease. Annu Rev Cell Dev Biol 20: 781–810. 15473860 10.1146/annurev.cellbio.20.010403.113126

[16] Clevers H (2006) Wnt/beta-catenin signaling in development and disease. Cell 127: 469–480. 17081971 10.1016/j.cell.2006.10.018

[17] Irizarry RA, Hobbs B, Collin F, Beazer-Barclay YD, Antonellis KJ, et al (2003) Exploration, normalization, and summaries of high density oligonucleotide array probe level data. Biostatistics 4: 249–264. 12925520 10.1093/biostatistics/4.2.249

[18] Siegfried E, Chou TB, Perrimon N (1992) wingless signaling acts through zeste-white 3, the Drosophila homolog of glycogen synthase kinase-3, to regulate engrailed and establish cell fate. Cell 71: 1167–1179. 1335365 10.1016/s0092-8674(05)80065-0

[19] Wang Y, Macke JP, Abella BS, Andreasson K, Worley P, et al (1996) A large family of putative transmembrane receptors homologous to the product of the Drosophila tissue polarity gene frizzled. J Biol Chem 271: 4468–4476. 8626800 10.1074/jbc.271.8.4468

[20] Wodarz A, Nusse R (1998) Mechanisms of Wnt signaling in development. Annu Rev Cell Dev Biol 14: 59–88. 9891778 10.1146/annurev.cellbio.14.1.59

[21] Huelsken J, Behrens J (2002) The Wnt signalling pathway. J Cell Sci 115: 3977–3978. 12356903 10.1242/jcs.00089

[22] Behrens J, von Kries JP, Kuhl M, Bruhn L, Wedlich D, et al (1996) Functional interaction of beta-catenin with the transcription factor LEF-1. Nature 382: 638–642. 8757136 10.1038/382638a0

[23] Gordon MD, Nusse R (2006) Wnt signaling: multiple pathways, multiple receptors, and multiple transcription factors. J Biol Chem 281: 22429–22433. 16793760 10.1074/jbc.R600015200

[24] Carrero JJ, Yilmaz MI, Lindholm B, Stenvinkel P (2008) Cytokine dysregulation in chronic kidney disease: how can we treat it? Blood Purif 26: 291–299. 18421214 10.1159/000126926

[25] Silverstein DM (2009) Inflammation in chronic kidney disease: role in the progression of renal and cardiovascular disease. Pediatr Nephrol 24: 1445–1452. 19083024 10.1007/s00467-008-1046-0

[26] Recio-Mayoral A, Banerjee D, Streather C, Kaski JC (2011) Endothelial dysfunction, inflammation and atherosclerosis in chronic kidney disease–a cross-sectional study of predialysis, dialysis and kidney-transplantation patients. Atherosclerosis 216: 446–451. 21414625 10.1016/j.atherosclerosis.2011.02.017

[27] Heine GH, Ortiz A, Massy ZA, Lindholm B, Wiecek A, et al (2012) Monocyte subpopulations and cardiovascular risk in chronic kidney disease. Nat Rev Nephrol 8: 362–369. 22410492 10.1038/nrneph.2012.41

[28] Rogacev KS, Seiler S, Zawada AM, Reichart B, Herath E, et al (2011) CD14++CD16+ monocytes and cardiovascular outcome in patients with chronic kidney disease. Eur Heart J 32: 84–92. 20943670 10.1093/eurheartj/ehq371

[29] Kikuchi A, Yamamoto H, Sato A, Matsumoto S (2012) Wnt5a: its signalling, functions and implication in diseases. Acta Physiol (Oxf) 204: 17–33. 21518267 10.1111/j.1748-1716.2011.02294.x

[30] Cadigan KM, Nusse R (1997) Wnt signaling: a common theme in animal development. Genes Dev 11: 3286–3305. 9407023 10.1101/gad.11.24.3286

[31] George SJ (2008) Wnt pathway: a new role in regulation of inflammation. Arterioscler Thromb Vasc Biol 28: 400–402. 18296599 10.1161/ATVBAHA.107.160952

[32] Pereira CP, Bachli EB, Schoedon G (2009) The wnt pathway: a macrophage effector molecule that triggers inflammation. Curr Atheroscler Rep 11: 236–242. 19361356 10.1007/s11883-009-0036-4

[33] Sen M, Lauterbach K, El-Gabalawy H, Firestein GS, Corr M, et al (2000) Expression and function of wingless and frizzled homologs in rheumatoid arthritis. Proc Natl Acad Sci U S A 97: 2791–2796. 10688908 10.1073/pnas.050574297 PMC16008

[34] Polzer K, Diarra D, Zwerina J, Schett G (2008) Inflammation and destruction of the joints–the Wnt pathway. Joint Bone Spine 75: 105–107. 18313347 10.1016/j.jbspin.2007.10.005

[35] Bienz M (2005) beta-Catenin: a pivot between cell adhesion and Wnt signalling. Curr Biol 15: R64–67. 15668160 10.1016/j.cub.2004.12.058

[36] Katoh M, Katoh M (2007) STAT3-induced WNT5A signaling loop in embryonic stem cells, adult normal tissues, chronic persistent inflammation, rheumatoid arthritis and cancer (Review). Int J Mol Med 19: 273–278. 17203201

[37] Katoh M, Katoh M (2009) Transcriptional mechanisms of WNT5A based on NF-kappaB, Hedgehog, TGFbeta, and Notch signaling cascades. Int J Mol Med 23: 763–769. 19424602 10.3892/ijmm_00000190

[38] Pereira C, Schaer DJ, Bachli EB, Kurrer MO, Schoedon G (2008) Wnt5A/CaMKII signaling contributes to the inflammatory response of macrophages and is a target for the antiinflammatory action of activated protein C and interleukin-10. Arterioscler Thromb Vasc Biol 28: 504–510. 18174455 10.1161/ATVBAHA.107.157438

[39] Lehtonen A, Ahlfors H, Veckman V, Miettinen M, Lahesmaa R, et al (2007) Gene expression profiling during differentiation of human monocytes to macrophages or dendritic cells. J Leukoc Biol 82: 710–720. 17595377 10.1189/jlb.0307194

[40] Chaussabel D, Semnani RT, McDowell MA, Sacks D, Sher A, et al (2003) Unique gene expression profiles of human macrophages and dendritic cells to phylogenetically distinct parasites. Blood 102: 672–681. 12663451 10.1182/blood-2002-10-3232

[41] Valencia J, Hernandez-Lopez C, Martinez VG, Hidalgo L, Zapata AG, et al (2011) Wnt5a skews dendritic cell differentiation to an unconventional phenotype with tolerogenic features. J Immunol 187: 4129–4139. 21918189 10.4049/jimmunol.1101243

[42] Tickenbrock L, Schwable J, Strey A, Sargin B, Hehn S, et al (2006) Wnt signaling regulates transendothelial migration of monocytes. J Leukoc Biol 79: 1306–1313. 16565323 10.1189/jlb.0905539

[43] Lee DK, Nathan Grantham R, Trachte AL, Mannion JD, Wilson CL (2006) Activation of the canonical Wnt/beta-catenin pathway enhances monocyte adhesion to endothelial cells. Biochem Biophys Res Commun 347: 109–116. 16815294 10.1016/j.bbrc.2006.06.082

[44] Kim J, Kim J, Kim DW, Ha Y, Ihm MH, et al (2010) Wnt5a induces endothelial inflammation via beta-catenin-independent signaling. J Immunol 185: 1274–1282. 20554957 10.4049/jimmunol.1000181

[45] Christman MA, 2nd, Goetz DJ, Dickerson E, McCall KD, Lewis CJ, et al (2008) Wnt5a is expressed in murine and human atherosclerotic lesions. Am J Physiol Heart Circ Physiol 294: H2864–2870. 18456733 10.1152/ajpheart.00982.2007

